# Perinatal Borderline Personality Disorder with Suicidality: An Integrative Treatment Approach in Two Case Reports

**DOI:** 10.1192/j.eurpsy.2026.12040

**Published:** 2026-07-31

**Authors:** M. Marković, D. Mladenović, M. Gojković, D. Mušić, B. Savić, S. Pejović Nikolić

**Affiliations:** 1 Clinic for Psychiatry, University Clinical Centre of Serbia, Belgrade; 2 Special Hospital for Psychiatric Disorders “Kovin”, Kovin; 3 Department for psychiatry, General Hospital Novi Pazar, Novi Pazar; 4 School of Medicine, University of Belgrade, Belgrade, Serbia

## Abstract

**Introduction:**

Borderline personality disorder (BPD) is a complex and clinically challenging condition, particularly during pregnancy and the postpartum. Pregnancy may exacerbate emotional instability, and while no drug is specifically approved for BPD, there is a huge unmet need for its effective treatment. Pregnant women with BPD are at elevated risk of complications, with potential adverse effects on child development. Suicidality in perinatal BPD is a major challenge, as the perinatal period is a time of heightened vulnerability. In BPD, where subjective perceptions strongly influence outcomes, patient perspectives inform a more patient-centered approach. This report presents two cases illustrating an integrative treatment model tailored to BPD symptoms in the peripartal period.

**Objectives:**

The aim of this report is to demonstrate the application of an integrative treatment approach for suicidal patients with borderline personality disorder during the peripartal period.

**Methods:**

Two cases are described. Patient 1 was a 35-year-old multiparous woman with BPD and chronic insomnia, presenting with suicidal ideation at 21 weeks. Patient 2 was a 19-year-old primiparous woman with BPD who presented with suicidal ideation at 14 weeks. Both had histories of severe trauma and limited social support. Each was followed for six months postpartum. Treatment combined pharmacotherapy (quetiapine, lamotrigine, benzodiazepines) and supportive psychodynamic psychotherapy. Both used innovative infant-sleep technology. Depression and anxiety were assessed with HAMD and HAMA, sleep with PSQI, and patient perspectives with a tailored questionnaire.

**Results:**

Both patients achieved resolution of suicidality and symptomatic improvement, though trajectories differed. Patient 1 remained in the mild-to-moderate depression range (mean HAMD 13.2, range 8–22), peaking at day 100 postpartum (22) before improving to 8 by six months. Anxiety fluctuated mildly to moderately (HAMA 6–15; mean 10). Sleep was persistently poor (PSQI 12–18). She rated treatment components as very to extremely helpful. Patient 2 initially improved (HAMD 6–9, HAMA 6–9), but after 120 days postpartum symptoms worsened to moderate levels (HAMD 17, HAMA 21 by day 175), with parallel sleep deterioration (PSQI 11–12). She rated treatment as moderately helpful overall. Both delivered healthy term infants, maintained bonding, and provided adequate care.

**Conclusions:**

These cases illustrate distinct perinatal BPD trajectories: one with early persistent depression and insomnia, another with delayed postpartum worsening. Both benefited from an integrative treatment approach that stabilized maternal functioning and preserved mother–infant bonding. Findings emphasize the need for comprehensive, patient-centered, multimodal interventions and highlight the value of incorporating patient-reported outcomes alongside clinician ratings.

**Disclosure of Interest:**

None Declared

